# The pyramid model as a structured way of quality management

**DOI:** 10.4103/0973-6247.39503

**Published:** 2008-01

**Authors:** Willem PA van der Tuuk Adriani, Smit Sibinga

**Affiliations:** *Academic Institute for International Development of Transfusion Medicine, University of Groningen, the Netherlands*

**Keywords:** Pyramid model, quality system, quality assurance, Good Manufacturing Practice (GMP), ISO 9001, GMP for blood banks, quality policy and strategy

## Abstract

Three quality systems that can be used in blood establishments are briefly explained. The Pyramid model is described as a tool to manage the quality systems. Finally, some experiences in other countries are given to prove the validity of the system.

## Introduction

Before the industrial revolution, craft workers made their products and only those products that fulfilled their requirements were sold (100% quality control). With the introduction of bulk production, 100% quality control became quite expensive and no longer feasible. Statistical quality control was introduced.

With the increase of product reliability, the system of quality assurance or Good Manufacturing Practice (GMP)[1] was introduced for the pharmaceutical industry; later, a generic quality system (ISO 9001:2000) was developed,[2] including the involvement of external parties or stake holders such as customers and general public.

In many countries, by law, the pharmaceutical industry must comply with the requirements of GMP and they are regularly inspected by a competent authority such as the FDA.

Blood products consist of living cells. As they are used for therapeutic purposes, they must be produced in accordance to the pharmaceutical production rules. This includes the introduction of the quality system Good Manufacturing Practice (GMP), originally developed by FDA for the pharmaceutical industry. Not all the requirements of the International GMP apply to blood banks. Therefore, the Dutch blood bank organization Sanquin developed the Sanquin guideline “GMP for blood banks.”[3] Recently, the European Union (EU) has accepted the Directive 2005/62/EU[4] containing the quality requirements for blood establishments. However, this quality system is product-oriented and not all departments of a Blood Centre or Blood Establishment are involved in this quality system. Therefore, it is advisable to look at other quality systems such as ISO 9001 and the European Foundation of Quality Management (EFQM).[5,6] With the current version of ISO 9001 issued in 2001, most of the specific elements of EFQM are now part of ISO 9001:2000. However, EFQM contains a specific method of self-auditing by analyzing the various phases of the departments or processes of an organization (see below).

The strong points of ISO 9001:2000 are management involvement (annual management review is required) and supplier chain (supplier - producer - customer (internal or external)) and customer satisfaction.

Until the development of quality assurance (QA) principles, there was no real management of the system. Operational documents such as SOPs (Standard Operating Procedures) and other documents were generated if necessary. This resulted in the following two problems:

1. Is the documentation complete?
2. How to write a quality manual?

The EFQM is actually not a quality system but a management system. It recognizes five different development stages of quality management:

1. Activity-or product-oriented: the system is restricted to SOPs and EOPs and related records and forms. The focus is on quality control (QC)
2. Process-oriented: SOPs and EOPs are more cohesive as the focus is on quality assurance (QA).
3. System-oriented: processes are cohesive and the focus is on GMP.
4. Chain-oriented: suppliers and customers are involved in the quality system, which has now become ISO based
5. Recognized for excellence: results and employees are involved in the quality system and the focus is on holistic management of all the aspects.

The Pyramid model includes all these phases.

## The Pyramid Concept

**Figure 1:**
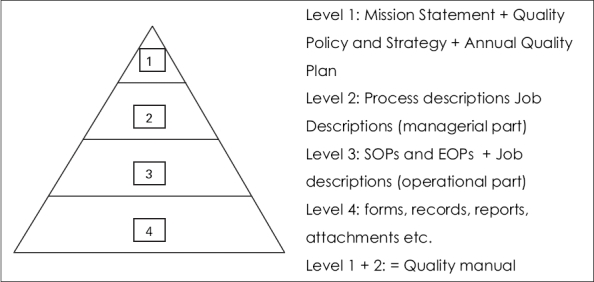
The Pyramid.

The Pyramid model consists of four layers or levels [Figure 1].

First of all, Level 1 contains the Mission Statement. This statement describes the ultimate goals and objectives of the Blood Establishment. This statement must be approved and authorized by the top management of the organization and where appropriate by the Ministry of Health. However, it can be developed by a broad group of coworkers of the organization. This is very helpful in obtaining a high level of commitment within the organization and a good relation with the customers if they are involved in the setting-up of the statement. This can be done by organizing a meeting with members of the middle management, representative(s) of hospital(s) and chaired by the CEO. People are asked to identify goals, activities, ideas, etc. Later, all these items can be translated into the mission statement that must be brief and comprehensive (maximum 1 or 2 sentences); it should be valid for at least 5-10 years. Consequently, these goals must appear in the organogram as the core business. For instance, in the Mission Statement, if the organization does not mention any medical advices given to the hospitals, a department for medical advices cannot be one of the core departments but a supporting department such as quality management, human resource management, personnel, and finance and administration.

The second element of the first level is Quality Policy and Strategy. This is the translation of the mission statement into the way how the goals and objectives have to be achieved. The CEO and the management team should prepare this document. The type of the organization must be described and an organogram must be included. All processes used in the various departments must be mentioned. These departments are the core departments (blood collection, processing, testing, storage and distribution, and medical advisory unit), the supportive departments (e.g., quality assurance or management, administration (secretary), human resource management and finance management) and the top management.

It is important that all the processes are mentioned in this document. Processes that are not mentioned here cannot be described in the next layer.

At least the following departments should be included:

Donor Affairs: donor management (information, motivation, retention of voluntary non-remunerated donors, the call-up system for blood donation and donor counseling), donor reception and identification, selection, treatment, blood collection, mobile blood collection, labeling, sampling and donor care during and after donation, and apheresis (if applicable).

Blood processing: centrifugation, primary processing, secondary processing, other processes such as leukocyte depletion, washing red cells, irradiation and where appropriate cryoprecipitation, (interim) labeling, product release, storage and distribution, temperature monitoring and evaluation.

Blood testing: blood grouping ABO/Rh(D), infectious disease marker testing and related algorithms, quality control of intermediates and final products and Immunohematology diagnostics (if appropriate).

Medical services: donor selection and clinical consultative services.

Research and development: (if appropriate and focused on improvement and development)

Quality department: quality system(s) (GMP, ISO, EFQM), document control, equipment control, stock control, non-conformance policy, complaints from donors, customers or suppliers, validation, internal audits, supplier audits, hygiene and safety, continuous improvement, monitoring and evaluation through statistical process control (SPC), risk management, safety and hygiene, proficiency testing, product release (final products and incoming critical goods).

CEO: Tasks, authorities, responsibilities, accountability, annual report, management review.

Human resource management or Personnel department: personnel management, training and assessment, schemes for personnel, salary administration.

Financial department: financial organization and structure, primary purchasing (together with the Quality Manager and the head of Departments), etc.

Stock management: routine purchasing, incoming goods inspection, quarantine of critical incoming goods and stock control.

Medical department: donor selection, donor care and advising hospital clinicians

Finally, this level contains the annual quality plan. The quality manager, supported by the CEO, should prepare this document. It must contain the plans for the following or current year in terms of the development and improvement of the quality system(s). The document must contain measurable objectives in terms of quality and time schedule. At the end of each period, the quality manager should write an evaluation report and it should have good arguments and explanations for not achieving one or more of the objectives (root cause analysis).

Level 2 contains the process descriptions, including the basic performance requirements of personnel (managerial part of job description). The processes mentioned in the quality policy and strategy must be described in detail. The principle and scope of the process, abbreviations used and definitions of words with a special meaning of uncommon words, tasks, authorization, responsibilities and accountability of all employees involved in the process, list of related processes and the final description of the process (including all the Standard Operating Procedures (SOPs) and Equipment Operating Procedures (EOPs) needed to perform the process) and list forms or records required to document the outcomes of the activities performed.

These process descriptions should be prepared by the heads of departments with, where necessary, the help of the operational staff.

Job descriptions (JD), such as operating procedures, consists of two parts: the managerial part (name and description of the function, required education (level) and accountability) and the list of specific tasks, which is part of level 3.

Level 1 and 2 together contain all the elements that must be present in the quality manual. Therefore, time can be saved because the quality manual need not be written separately as it already exists in its elements.

Level 3 contains the SOPs, EOPs and JDs (operational or functional part). Once the processes have been extensively described, the work instructions of how to perform a procedure or how to handle equipment can now easily be written by the operators who perform the procedures or operate the equipment. As a consequence, the language in which the work instructions are to be written should be clear to those who need to understand and operate the procedure and/or equipment.

SOPs and EOPs contain as a minimum an introduction and the work instructions. Where necessary and/or appropriate, some tasks responsibilities and authorization can be repeated from the process description(s).

The job descriptions should have at least the following subjects:

Name of the organization, name of the department, title of the function, educational/vocational requirements or level, salary scale, short description of the organization, department and function and tasks, authorities, responsibilities and accountability.

For each and every function, such a description must be available and it can be used for creating an advertisement for the new employees and can be used during the interview with candidates for a defined function.

Finally, there is level 4 that contains all the level-3-related forms, reports, records, attachments, operating manuals, etc.

As quality management systems require to document each and every activity, it must be done in a structured manner. Small pieces of paper will easily disappear and the documentation is lost. Appropriate and identifiable forms, records, reports etc. are therefore necessary.

The four levels of the Pyramid model translated into the EFQM phases of development could be presented as follows:

**Level 1** - this is the level of Excellence and transformation phase into the System and Chain phases.

**Level 2** - This level is the Process phase

**Level 3** - This level is the Activity or Process phase

**Level 4** - This is the ultimate first Product phase

## Discussion

In several countries, this model is discussed and introduced. The reactions are quite different, for instance, consider the following.

The North Estonian Blood Bank considered the system as an eye-opener, and it was introduced to support their existing quality system.

In some countries in Africa and Eastern Europe, it seemed to be more difficult to transfer the theory into practice. People reported to have fully understood the system, although they continued writing their SOPs in the old and traditional way. In these countries, more intensive training is required to achieve the right perception of the principle.

The model was also introduced in a Hospital Blood Bank in Nicosia. The system was very well understood and implemented after complete explanation. Two more short sessions followed and process analysis revealed that some SOPs were missing.

Practice has now shown that the model really works well. The system proves that documentation is complete to the level of process description.

## Conclusion

The pyramid model is a clear and well functioning system to manage the required quality documentation at all levels - managerial and operational. It is simple, practical, enables to prove that the documentation is complete, and provides and maintains the quality manual.
